# Diffuse Large B-Cell Lymphoma of the Mandible Diagnosed by Metagenomic Sequencing: A Case Report

**DOI:** 10.3389/fmed.2021.752523

**Published:** 2021-12-23

**Authors:** Kaili Liu, Yang Gao, Jiawei Han, Xu Han, Yingqi Shi, Chao Liu, Jie Li

**Affiliations:** ^1^Department of General Medical Ward, First Affiliated Hospital of Soochow University, Suzhou, China; ^2^School of Biological Science and Medical Engineering, Beihang University, Beijing, China; ^3^Department of Neurosurgery, The Second Affiliated Hospital, Zhejiang University School of Medicine, Hangzhou, China; ^4^Department of Medical, Hangzhou Matridx Biotechnology Co., Ltd, Hangzhou, China

**Keywords:** non-Hodgkin lymphoma, mandibular NHL, diffuse large B-cell lymphoma, copy number variations, metagenomic next-generation sequencing, fever of unknown origin

## Abstract

**Introduction:** Non-Hodgkin lymphoma (NHL) has a much higher incidence rate than Hodgkin lymphoma. Approximately 40% NHL occurs in extranodal tissues or organs, and its clinical manifestations are often nonspecific. Primary bone NHL involving the mandible is an uncommon NHL that is characterized by fever, gum swelling and toothache. Therefore, it is often misdiagnosed as oral diseases.

**Case Presentation:** A 52-year-old female had recurrent fever for more than 1 month, with numbness in her left jaw and toothache. PET/CT showed an uptake area in the left mandible, suggesting microbial infections. However, antibacterial, and antiviral treatment were ineffective. Furthermore, metagenomic sequencing of plasma reported no pathogens, but instead showed significant copy number variations of multiple chromosomes, which highly suggested the existence of tumor. Finally, diffuse large B-cell lymphoma (DLBCL) was diagnosed by mandibular biopsy, and the patient was transferred to Hematology department for chemotherapy.

**Conclusion:** mNGS not only assists rapid etiological diagnosis, but also helps rule out infection and diagnose malignant neoplasm.

## Introduction

Lymphomas are a group of malignant neoplasms originated from lymphoid hematopoietic system and the second most common malignancy of the head and neck region (1–3). Hodgkin lymphoma (HL) and non-Hodgkin lymphoma (NHL) are the two main categories of lymphomas, the latter of which occurs more frequently (4). NHL patients usually present with painless lymphadenopathy, and might or might not have other symptoms including fevers, fatigue and drenching night sweats (5). NHL is primarily a disease of the lymph nodes, but it can also involve organs outside the lymphoid system such as the stomach, skin, lung, central nervous system, and oral cavity (6). Primary bone NHL involving the mandible, an uncommon oral cavity NHL, accounts for 0.6% of extranodal lymphomas (7). The clinical manifestations of Primary bone NHL involving the mandible are toothache, swelling, tooth loosening and pathological fracture. In early stage, it is often misdiagnosed as dental pulp disease, causing a delay in the diagnosis and treatment (7). Most Primary bone NHL involving the mandible are diffuse large B-cell lymphoma (DLBCL), which develops rapidly and has poor prognosis (8).

Metagenomic Next-generation Sequencing (mNGS) can unbiasedly sequence nucleic acids of both human and microbial origin from clinical samples. It is a hypothesis-free and culture-free technique that has been widely used in diagnosing infectious diseases (9). A retrospective study of patients with fever of unknown origin showed that the positive rate of mNGS in plasma samples was significantly higher than traditional culture (61.0 vs. 19.5%) (10). Meanwhile, the sequences of human DNA derived from mNGS can be used to detect chromosomal copy number variations, which might be useful for the diagnosis of cryptogenic malignancies (11).

Here we report a case of mandibular DLBCL in a 52-year-old female with recurrent fever and toothache, in which microbial infections were excluded based on mNGS results. Moreover, cancer was suspected due to abnormal copy number of the patient's chromosomes via analysis of host DNA sequences, which was later confirmed by mandible biopsy.

## Case Report

### Case Presentation

**Figure 1:**
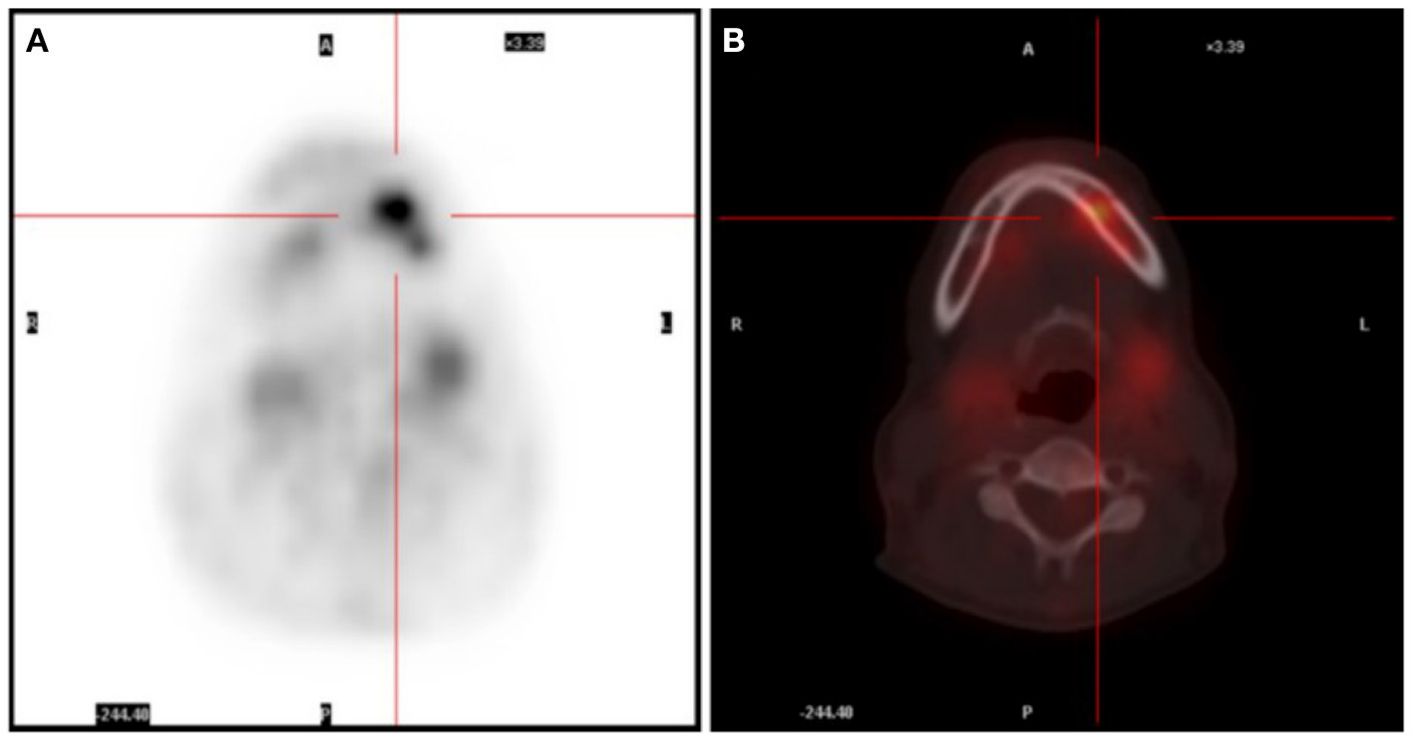
**(A)** PET scan showing an area with glucose uptake in the facial region. **(B)** PET/CT scan revealing the uptake area located in the left mandible.

A 52-year-old female presented with recurrent fever for more than 1 month, with a highest temperature of 38.8°C and chills. The patient experienced numbness in her left mandible and slight pain in her left teeth. The blood routine examination was normal, C-reactive protein (CRP) was 7.33 mg/L, erythrocyte sedimentation rate (ESR) was 42 mm/h, rheumatoid factor was 5.7 IU/ml, anti-o was 89 IU/ml, anti-nuclear antibody, alpha work, Epstein Barr virus, cytomegalovirus DNA, tumor biomarkers were negative. The head (including the mandible region), chest, and abdomen CT showed no active lesions, and echocardiography showed no abnormality. Prior to hospitalization, the patient had a full-body positron emission tomography (PET) scan performed, which revealed an elevated glucose metabolism in the left alveolar branch of the mandible (standardized uptake value (SUV) max = 5.87) (Figure 1). In addition, multiple small nodules were seen in both lungs and exudative changes in the lower lobes of both lungs and splenomegaly were observed. These findings were also confirmed by chest, abdomen, and pelvis enhanced CT. As a result, microbial infection was primarily suspected. Following 4 weeks of antibacterial and antiviral treatment, the patient still had recurrent fever, which was accompanied by perspiration. The blood routine showed the red blood cell count was 3.10^*^10^12^/L, hemoglobin was 86 g/L, neutrophil was 1.77^*^10^9^/L, lymphocyte was 0.71^*^10^9^/L, CRP was 16.6 mg/L.

Urine routine examination showed that urine occult blood was weakly positive. Stool routine, blood transfusion routine, blood culture, urine culture, humoral immunity, Widal test, Ana antibody spectrum, antineutrophil cytoplasmic antibody, G test, and EBV-DNA were negative. GM test (0.59) and T-spot.TB test were positive. The biochemical examination showed normal liver and kidney function, albumin 26.7 g/L, ESR 46 mm/h, coagulation function showed D-Dimer was 0.73 mg/L, and prothrombin time was 15.30 s. Tumor biomarker test showed ferritin was 363.36 ng/ml, and serum immunofixation electrophoresis was negative. Ultrasound examination showed no obvious abnormalities of superficial lymph nodes, esophagus, and heart. However, abdominal ultrasound showed substantial echo at the lower pole of spleen, and accessory spleen was considered.

### Initial Treatment

The patient was first administered with ticarcillin and moxifloxacin. Nine days after treatment, the fever was still present that peaked at 38.6°C. The blood routine and biochemical examination showed no apparent changes. The serum protein electrophoresis and free light chain quantitative were negative. The antibiotics were then escalated to linezolid combined with sulperazon and tinidazole. Three days later, the patient's symptoms did not improve, and thus antibiotics were withdrawn.

### MNGS Testing

**Figure 2:**
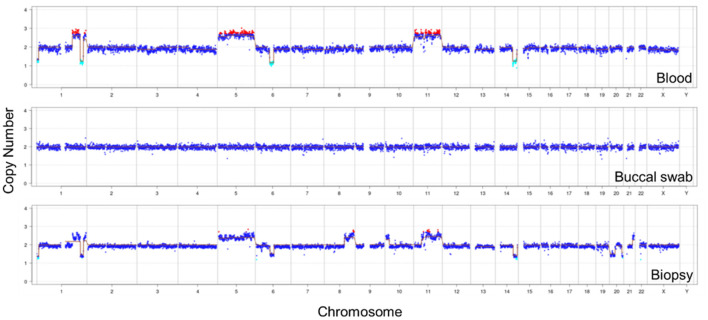
CNV analysis of the patient's autosomes and sex chromosomes. Both plasma and mandibular biopsy samples showed large CNVs on chromosomes 1, 5, 6, 11, and 14. The same analysis using buccal swab showed normal copy numbers.

Due to little effect of antibiotic treatment, the patient's peripheral blood was collected for mNGS. No pathogen was detected, suggesting a likelihood of non-infectious disease. Meanwhile, the chromosomal copy number analysis showed significant alterations (both duplications and deletions) in chromosomes 1, 5, 6, 11, and 14, which indicated the presence of malignant tumor DNA (Figure 2). The same CNV analysis was repeated using buccal swab that showed no abnormalities, suggesting the CNVs were not due to inherited diseases.

### Follow-Up and Outcomes

**Figure 3:**
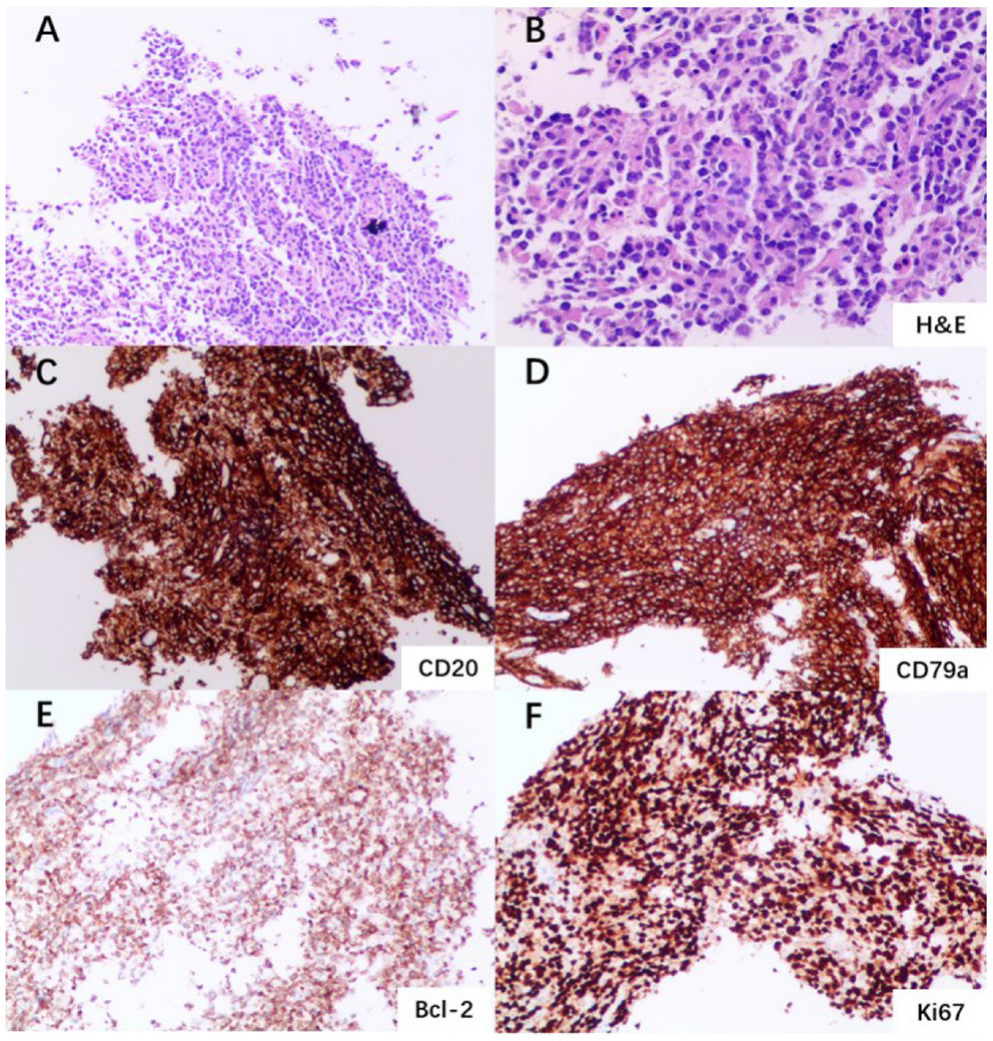
Left mandible biopsy and immunohistochemistry. **(A)** Multiple fragments of a squamous mucosa and soft tissue with a dense lymphoid infiltrate (×100 magnification). **(B)** Aggregate of atypical lymphoid cells (H&E staining; ×200 magnification). **(C)** Immunohistochemical staining showing sheets of large mononuclear lymphoid cells positive for CD20 (×100 magnification). **(D)** Sheets of large mononuclear lymphoid cells positive for CD79a (×100 magnification). **(E)** Cells positive for Bcl-2 staining (×100 magnification). **(F)** Cells positive for Ki67 staining (×100 magnification).

The patient underwent bone marrow biopsy. The morphological examination showed that the percentage of erythroid cells was slightly higher, and iron staining showed that iron utilization was impaired. Hemophagocytes were sporadically seen in the bone marrow. A biopsy of the left mandible was taken, and the immunohistochemical results showed cells positive for CD20, CD79a, Bcl-2, CD5 and c-myc, and Ki-67 value-added index was 90% (Figure 3). In addition, CD10, kappa, lambda, CD138, CD38, CD3, Desmin, MyoD1, Myogenin, CD99, CK, Syn, EMA, CyclinD1, Bcl-6, MUM1, CD30 and Sox11 were negative. The pathological findings suggested that the tumor was malignant B-cell lymphoma (diffuse large B-cell type). The patient was diagnosed with DLBCL and transferred to the Hematology department for immunochemotherapy [rituximab 600 mg d1, cyclophosphamide 1.1 g d2, doxorubicin 40 mg d2, vindesine 4 mg d2, and prednisone 15 mg d1-5 (R-CHOP)]. Five cycles of R-CHOP were administered and another full-body PET-CT was performed, which showed a decline of glucose metabolism in the left mandible region (Supplementary Figure 1). Adjuvant radiation therapy was initiated following the conclusion of chemotherapy and a total of 15 sessions were performed, after which the patient was discharged. During the most recent follow up visit, the patient had no fever, no cough, no abdominal pain, normal diet, and sleep.

## Discussion

Approximately 40% of NHL occurred in extranodal tissues or organs (12, 13), and the most common sites of occurrence was gastrointestinal tract, followed by head and neck (14). A retrospective study revealed that of 381 patients with NHL (100 were extranodal), 16 (16%) had maxillary or mandibular involvement and most of which were DLBCL (15). DLBCL is the most common type of NHL, accounting for 40% of all cases. It is a type of cancer involving distinct clinical, morphological, immunohistochemical features and prognosis (7). Primary bone NHL involving the mandible is often characterized by swelling, pain, abnormal sensation, loose teeth, and pathological fracture. Patients are usually treated in the Department of Stomatology and often misdiagnosed with oral diseases. In this case, the patient received dental root canal treatment and anti-infection treatment with negligible improvement of symptoms.

Fever of unknown origin can be attributed to many factors, including infection (40%), neoplastic disease (20%), collagen-vascular disease (15%) and so on (16). A retrospective study found that tuberculosis was the most common cause of infectious fever and lymphoma was the most common cause of neoplastic fever (17). In this case, the hemogram and blood biochemistry of the patient were basically normal, CRP and ESR were slightly elevated, indicating inflammatory reaction. Routine etiological tests were negative except for T-spot.TB test. However, chest CT showed no signs of tuberculosis infection. And GM test was positive for the first time but negative when repeated. Combined with the lack of pathogens in mNGS results, microbial infection was mostly ruled out. In addition, the full-body PET scan showed increased glucose metabolism in the left mandible, and ferritin and tumor specific growth factor were elevated in serum. However, tumor was not suspected prior to mNGS because abscess, inflammation and dental caries could also lead to increased glucose metabolism, and the specificity of tumor biomarkers was low.

mNGS has been predominantly used for diagnosing infectious diseases, even though most sequences in clinical samples originate from the host. By analyzing human reads, one could obtain potentially valuable information and use it to aid diagnosis.

Copy number variation (CNV) is the change of copy number in one or more genes. Genomic instability is one of the hallmarks of cancer (18). Multiple studies have shown that DNA copy number variation (CNV) is an important component of genetic variation involved in the development and progression of many types of cancer. Chromosomal CNVs detected by sequencing-based tests have been recently used for early diagnosis of colorectal cancer (19), breast cancer (20), and hematologic malignancies (21). Therefore, mNGS has the potential to diagnose both microbial infections and tumor since it unbiasedly sequences DNA of both host and microbial origin. In Cui et al. (22) detected CNV in clinical samples and cell lines of DLBCL patients, and the results showed that CNV was related to the prognosis of patients and might be an important feature of tumor development. Shotgun metagenomic sequencing is suitable for CNV detection across all chromosomes, which has good specificity and reproducibility (23).

The gold standard for tumor diagnosis is biopsy and pathological staining, which requires an invasive procedure that typically takes 3–7 days. In addition, biopsy is not always performed especially when tumor is not primarily considered. Compared to that, mNGS can be performed on a variety of clinical samples and report results in hours. Although the detection of chromosomal CNVs by mNGS cannot directly lead to a specific cancer diagnosis, it could prompt more focused diagnostic testing for a tumor. By incorporating pathogen detection and copy number analysis, mNGS can provide multiple levels of diagnostic clues. It helps optimize the sensitivity of tumor diagnosis and has significance in differential diagnosis for patients suspected of infections, especially when empirical antibiotic therapy fails.

## Data Availability Statement

The datasets presented in this study can be found in online repositories. The names of the repository/repositories and accession number(s) can be found below: https://www.ncbi.nlm.nih.gov/sra, accession number PRJNA752259.

## Ethics Statement

Written informed consent was obtained from the relevant individual(s), and/or minor(s)' legal guardian/next of kin, for the publication of any potentially identifiable images or data included in this article.

## Author Contributions

KL is the primary physician that provided diagnosis and treatment of the patient. YG collected and analyzed clinical and sequencing data. JH, XH, and YS performed metagenomic sequencing experiments and CNV analysis. CL and JL wrote the manuscript. All authors have read and approved the final version of the manuscript.

## Funding

This project was supported by the Science and Technology Plan Projects of Suzhou (grant no. SYS2020099).

## Conflict of Interest

XH, YS, and CL are employees of Hangzhou Matridx Biotechnology Co., Ltd. The remaining authors declare that the research was conducted in the absence of any commercial or financial relationships that could be construed as a potential conflict of interest.

## Publisher's Note

All claims expressed in this article are solely those of the authors and do not necessarily represent those of their affiliated organizations, or those of the publisher, the editors and the reviewers. Any product that may be evaluated in this article, or claim that may be made by its manufacturer, is not guaranteed or endorsed by the publisher.
