# Supplementary figures and images for: Diffuse Large B-Cell Lymphoma of the Mandible Diagnosed by Metagenomic Sequencing: A Case Report
Source: Front Med (Lausanne). 2021 Dec 23;8:752523. doi: 10.3389/fmed.2021.752523 (PMC8732773; doi:10.3389/fmed.2021.752523)

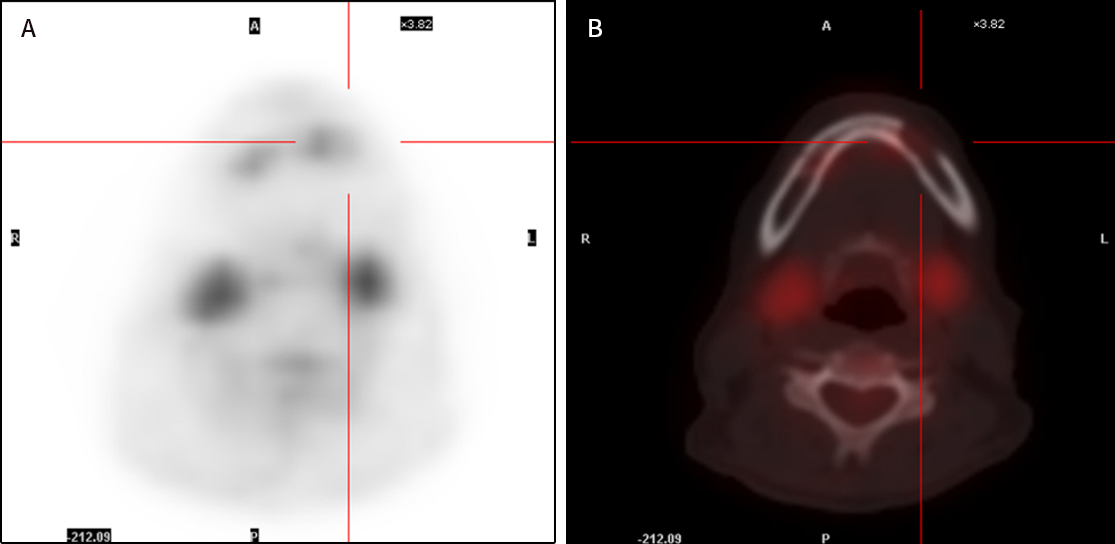

Supplement: Supplementary file 1 [file Image_1.TIF]
